# Magnetic Particle Imaging for High Temporal Resolution Assessment of Aneurysm Hemodynamics

**DOI:** 10.1371/journal.pone.0160097

**Published:** 2016-08-05

**Authors:** Jan Sedlacik, Andreas Frölich, Johanna Spallek, Nils D. Forkert, Tobias D. Faizy, Franziska Werner, Tobias Knopp, Dieter Krause, Jens Fiehler, Jan-Hendrik Buhk

**Affiliations:** 1 Department of Neuroradiology, University Medical Center Hamburg-Eppendorf, Hamburg, Germany; 2 Department of Product Development and Mechanical Engineering Design, Hamburg University of Technology, Hamburg, Germany; 3 Department of Radiology and Hotchkiss Brain Institute, University of Calgary, Calgary, Canada; 4 Section for Biomedical Imaging, University Medical Center Hamburg-Eppendorf, Hamburg, Germany; 5 Institute for Biomedical Imaging, Hamburg University of Technology, Hamburg, Germany; Technion - Israel Institute of Technology, ISRAEL

## Abstract

**Purpose:**

The purpose of this work was to demonstrate the capability of magnetic particle imaging (MPI) to assess the hemodynamics in a realistic 3D aneurysm model obtained by additive manufacturing. MPI was compared with magnetic resonance imaging (MRI) and dynamic digital subtraction angiography (DSA).

**Materials and Methods:**

The aneurysm model was of saccular morphology (7 mm dome height, 5 mm cross-section, 3–4 mm neck, 3.5 mm parent artery diameter) and connected to a peristaltic pump delivering a physiological flow (250 mL/min) and pulsation rate (70/min). High-resolution (4 h long) 4D phase contrast flow quantification (4D pc-fq) MRI was used to directly assess the hemodynamics of the model. Dynamic MPI, MRI, and DSA were performed with contrast agent injections (3 mL volume in 3 s) through a proximally placed catheter.

**Results and Discussion:**

4D pc-fq measurements showed distinct pulsatile flow velocities (20–80 cm/s) as well as lower flow velocities and a vortex inside the aneurysm. All three dynamic methods (MPI, MRI, and DSA) also showed a clear pulsation pattern as well as delayed contrast agent dynamics within the aneurysm, which is most likely caused by the vortex within the aneurysm. Due to the high temporal resolution of MPI and DSA, it was possible to track the contrast agent bolus through the model and to estimate the average flow velocity (about 60 cm/s), which is in accordance with the 4D pc-fq measurements.

**Conclusions:**

The ionizing radiation free, 4D high resolution MPI method is a very promising tool for imaging and characterization of hemodynamics in human. It carries the possibility of overcoming certain disadvantages of other modalities like considerably lower temporal resolution of dynamic MRI and limited 2D characteristics of DSA. Furthermore, additive manufacturing is the key for translating powerful pre-clinical techniques into the clinic.

## Introduction

Magnetic particle imaging (MPI) [1] has become a promising and innovative field of imaging research in the last decade. The fundamental property of MPI is its direct sensitivity to magnetic particles. This allows obtaining data with high contrast since no background signal from non-magnetic materials, like biological tissue, compromises the measurements.

Dedicated MPI tracers promise a high signal quality such that the method is potentially capable of detecting nanograms of iron [2], which is especially relevant for cell tracking applications [3]. Since MPI is further capable of acquiring 3D datasets with high temporal resolution [4], which can be used to detect a bolus of superparamagnetic particles flowing through the vessel system [5], it is predestinated for quantitative blood flow measurements as well as interventional applications where magnetic instruments can be visualized by an appropriate coating [6]. The characterization of the hemodynamics of aneurysms is of particular interest, since treatment planning and follow-up diagnosis may benefit from this new imaging technique [7–9]. The unique contribution of MPI to the clinical environment is its capability of quantitative high resolution 4D imaging without ionizing radiation. This will be very beneficial for many angiographic applications and especially endovascular interventionalists and their patients will greatly benefit from MPI, since they will not be exposed to ionizing radiation. Further advances in MPI, like differentiation of catheters and the vessel's lumen during intervention via colored MPI [10] or exerting forces to the tip of the angiography catheter, facilitating catheter advancement into the target vessel [11], will make MPI even more unique and appealing for clinical application.

To provide accurate, comparable and feasible measurements of pathologic flow characteristics in diseased vessels, many studies focused on the creation of ex vivo vessel models, providing distinct opportunities for the evaluation of implantable devices, practical training, or extraction and validation of hemodynamic characteristics [12–16]. Since no human MPI scanner is available up to date, patient specific additively manufactured (3D printed) aneurysm models are the key technology for translating MPI into the clinic. Therefore, the purpose of this work was to evaluate the concept of assessing the hemodynamics of a realistic aneurysm model with MPI and to compare the results with dynamic magnetic resonance imaging (MRI) and dynamic digital subtraction angiography (DSA).

## Materials and Methods

**Fig 1:**
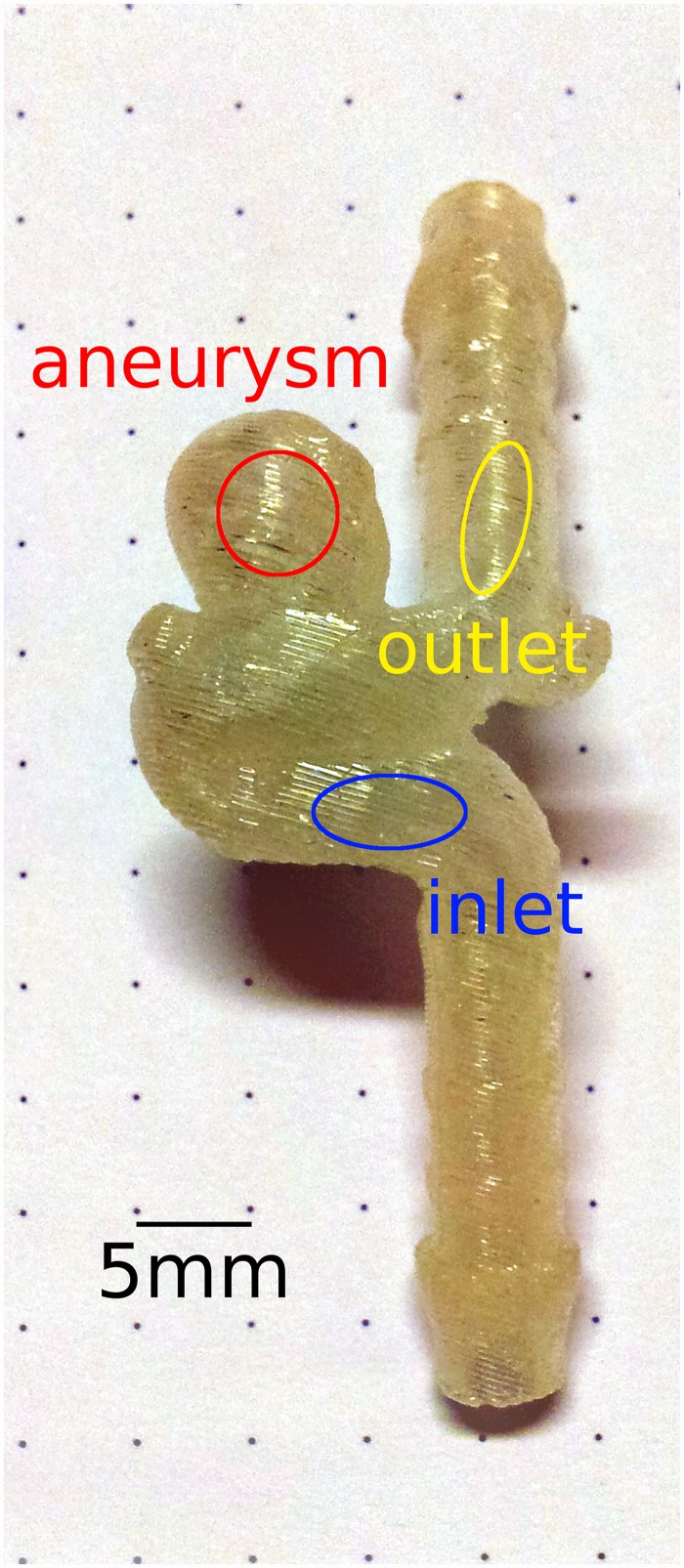
Photo of the aneurysm model. Regions or volumes of interest (inlet, aneurysm, outlet) were chosen at the indicated positions of the model in the corresponding 3D or 2D image data, respectively. The signal within these regions was plotted and analyzed to compare the imaging methods.

An additively manufactured realistic aneurysm model from a previous study [17] was used in our work. Thus, no patient specific data was available for our study. The aneurysm is located at the internal carotid artery (ICA) and of saccular morphology with 7 mm dome height, 5 mm cross-section, and 3–4 mm neck. The diameter of the parent artery was 3.5 mm. The model was manufactured with 254 μm thick layers of acrylonitrile butadiene styrene at fused deposition modeling, using the HP Designjet 3D printer (Hewlett-Packard Development Company, L.P., Palo Alto, CA, USA). At fused deposition modeling, a thermoplastic filament is liquefied and extruded through a nozzle. The machine deposits the filament along the extrusion path in multiple layers to build a three-dimensional part. Support structures were built where needed using a removable material, e.g. in the hollow vessel area. They were dissolved by using the Designjet 3D Removal System (Hewlett-Packard Development Company, L.P., Palo Alto, CA, USA). The layered structure of the model is leveled and smoothed to some extend by the transparent impregnation fluid Nano-Seal (Jeln Imprägnierung, Schwalmstedt, Germany) [17], not visible in the photograph (Fig 1), which seals pores and groves caused by the fused deposition modeling technique.

The regions of interest were placed in the parent vessel to be about the same distance from the neck of the aneurysm and to not extent into the artificial geometry of the tubes and tube connectors of the model. Therefore, the region upstream of the aneurysm is further away from the model inlet than the region downstream from the aneurysm is from the model outlet. Placing these regions was not based on clinical convention, but based on technical and methodical considerations to analyze and compare the flow pattern of the parent vessel before or after the aneurysm.

The aneurysm model was connected to a peristaltic pump (Sorin Group Deutschland GmbH, Munich, Germany), which was set to deliver a water flow with physiological flow and pulsation rate of about 250 mL/min and 70/min, respectively (S1 Fig). However, due to the peristaltic motion of the pump, the pulsation profile is not comparable with the pumping of a heart. The pulsating flow was running constantly for at least 10 minutes before each measurement eliminating initial disturbances or instabilities of the flow. The average flow velocity through the main vessel of the aneurysm model can be estimated by dividing the volume flow rate of the pump with the main vessel's cross section of the aneurysm model resulting in about 43 cm/s. Only an average flow velocity can be stated here, since we could only estimate the average flow by collecting the volume flowing into a graduated cylinder during one minute of time, i.e. 250mL/min. Measuring multiple flow velocities over the transient pulse waveform was only possible by using the 4D flow quantitative phase contrast MRI (Please see text below and the results section).

4D pc-fq MRI [18] and dynamic MRI, i.e. time-resolved contrast enhanced angiography with stochastic trajectories [19], were performed using a 7T small animal MRI (ClinScan, Bruker BioSpin MRI GmbH, Ettlingen, Germany) and a Bruker mouse body/rat head, quadrature transmit/receive volume coil. The 4D pc-fq sequence was triggered to be in sync with pump pulsation by placing the respiration sensor (pneumatic pillow) of a small animal monitor and gating system (SA Instruments, Inc., Stony Brook, NY) close to the tube outlet of the roller pump (S1 Fig). The total scan time of the 4D pc-fq measurement was 4 hours and 12 minutes to obtain sufficient high spatial resolution and signal to noise ratio. Further 4D pc-fq imaging parameters were summarized in Table 1.

**Table 1:** Detailed 4D pc-fq imaging parameters.

Contrast:	TE = 2.88 ms, TR = 17.75 ms, FA = 10°
Readout:	readout bandwidth = 941 Hz/pixel, strong asymmetric readout, 9 averages
Geometry:	FOV = 32x32x16 mm^3^, matrix = 64x64x32, 25% slice oversampling, 0.05 mm isotropic spatial resolution
Encoding:	46 phases, velocity encoding in 3 orthogonal directions with 122 cm/s

Imaging parameters for the dynamic MRI measurement were: TE = 0.5 ms, TR = 1.3 ms, FA = 50°, readout bandwidth = 1560 Hz/pixel and strong asymmetric readout, FOV = 23x23x6.88 mm^3^, matrix = 64x64x16, 50% slice resolution, 6/8 partial Fourier in both phase encoding directions, image resolution was reconstructed by k-space zero filling to 0.36x0.36x0.43mm^3^, temporal resolution was 270 ms, and 0.05 mol (Gd-DOTA)/L (Dotarem, Guerbet, Roissy CdG Cedex, France) was applied as contrast agent.

The contrast agent was administered by injecting a 3 mL bolus volume with a rate of 1 mL/s using a syringe pump and an angiographic catheter with 1 mm inner diameter. The tip of the catheter was placed close to the aneurysm model (about 5 cm upstream) to reduce bolus dispersion. The relatively slow injection rate was chosen to allow sufficient modulation of the contrast agent solution with the underlying pulsating flow in the main vessel [20]. Faster or slower injection rates will result in suboptimal contrast agent concentration modulation and, thus, were not investigated in our study. The bolus injection was identical for all dynamic imaging methods.

The first commercially available pre-clinical MPI scanner (Bruker Biospin GmbH, Ettlingen, Germany) [21] was used to acquire 1 mm isotropic 3D data with 21.54 ms temporal resolution while administering a bolus with 50 mmol(Fe)/L (MM4, micromod Partikeltechnologie GmbH, Rostock, Germany) similarly as for the dynamic MRI measurement. The selection gradient field of the MPI scan was 2.5  T/m, the amplitude of the drive field = 14 mT resulting in a FOV of 22.4x22.4x11.2 mm^3^. MPI requires a calibration measurement for image reconstruction, which is obtained by shifting a small voxel-shaped particle sample to all positions within the FOV. In order to prevent artifacts at the FOV boundaries [22], the calibration scan is captured in a larger volume of 32x32x18 mm^3^.

Dynamic DSA was acquired using an AlluraClarity Xper FD20 angiography system (Philips Healthcare, Best, Netherlands) during bolus injection of 150 mg(iodine)/mL (Imeron, Bracco-Altana, Konstanz, Germany) similarly as for the dynamic MRI and MPI measurements. DSA imaging parameters were: frame rate = 30/s yielding a temporal resolution of 33.33 ms, detector FOV = 15x15 cm^2^, image matrix = 1024x1024, X-ray tube current = 110 mAs, and voltage = 60 kVp.

All image post processing, quantitative analysis, and visualization was done with in house written software scripts using MATLAB (The Mathworks, Natick, MA, USA). For quantitative analysis of the dynamic imaging methods, the time difference between the points of the local maximum contrast agent concentration and the beginning and end of the bolus (approx. 25% of maximal signal/attenuation) were visually marked and analyzed. For bolus length and pulsation rate, the average time difference over all regions of the aforementioned time points of the contrast agent concentration curve were calculated. For the delay of the contrast agent dynamics in the aneurysm, the average time difference between the inlet and aneurysm region were calculated over all aforementioned time points of the contrast agent concentration curve.

## Results

**Fig 2:**
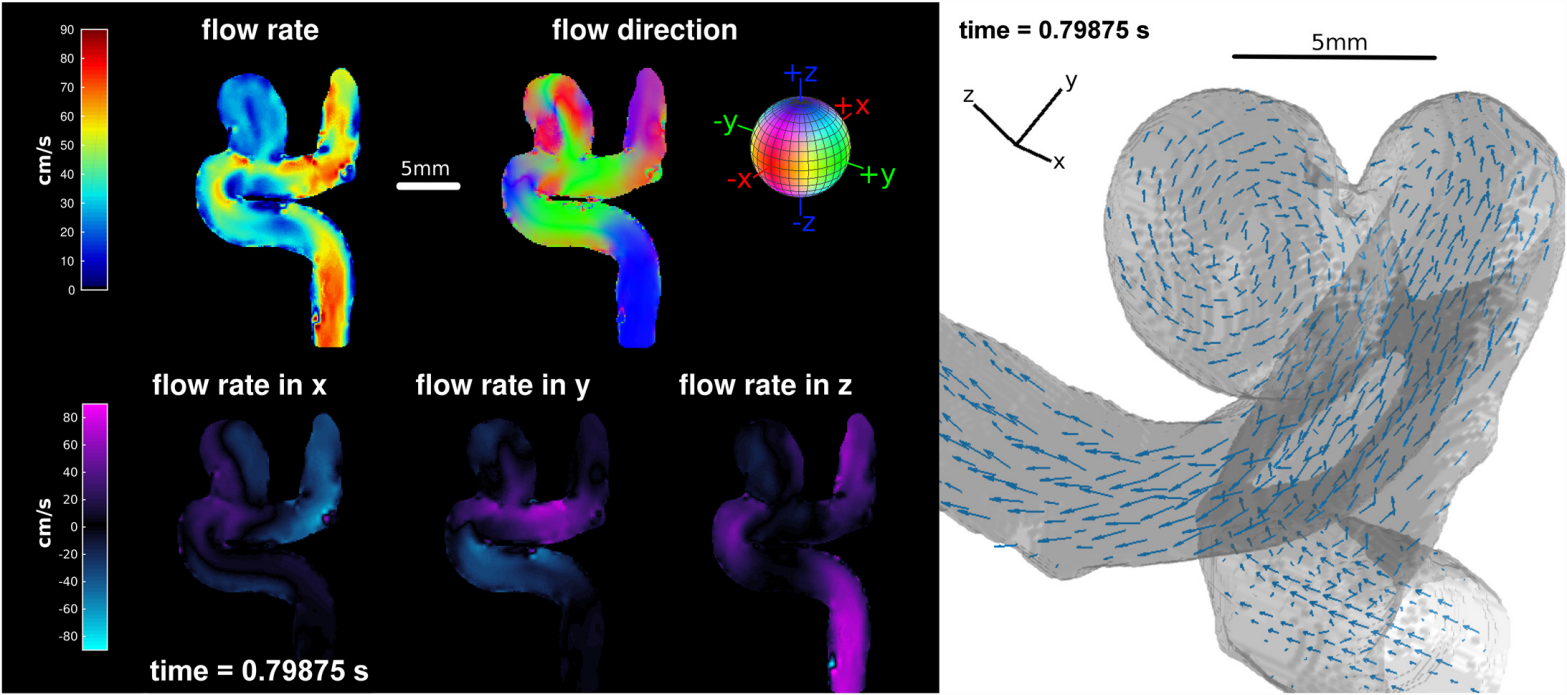
False color (left) and vector field (right) visualization of the 4D pc-fq MRI at the time point of maximal flow velocity during one pulsation cycle. Lower flow velocity and different flow directions are clearly visible inside the aneurysm. Movies showing all phases of the pulsation are available as S1 and S2 Videos.

**Fig 3:**
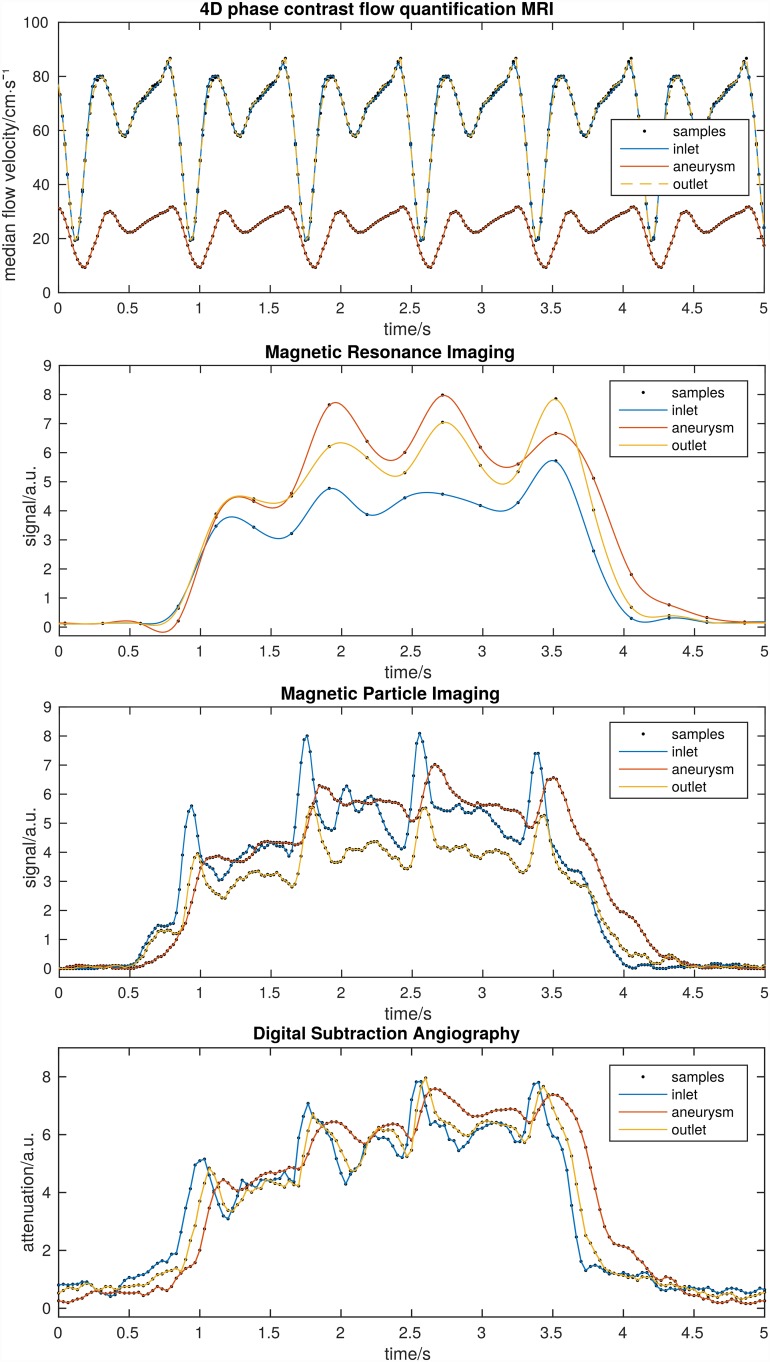
Cyclic repetition of the 4D pc-fq MRI (top) shows distinct pulsation and a much lower flow velocity inside the aneurysm. Dynamic MRI (second top, cubic spline interpolated), MPI (second bottom), and DSA (bottom) also show distinct pulsation and delayed contrast agent dynamics inside the aneurysm. Regions were chosen in the corresponding image data as depicted in Fig 1. Pulsations and bolus injections did not perfectly match between the different methods, due to the resetting of the experiments.

Distinct pulsatile flow as well as lower flow velocities and a vortex inside the aneurysm were clearly depicted by 4D pc-fq MRI (Figs 2 and 3). The flow velocity averaged over the pulsation cycle inside the main vessel of the model was 64±18 cm/s, which is higher than the expected flow velocity based on the average pump flow rate and vessel diameter (43 cm/s). This is caused by the laminar flow profile and by the regions of interest that cover more the faster flowing center of the main vessel. The flow velocity inside the aneurysm averaged over the whole pulsation cycle was 24±7 cm/s, which is much lower compared to the velocity in the main vessel of the model. Furthermore, the flow velocities of the model's inlet and outlet change simultaneously over the pulsation cycle, whereas the change of the flow velocity is clearly delayed inside the aneurysm.

Dynamic MPI, MRI, and DSA also showed a clear pulsation with higher signal or attenuation, i.e. contrast agent concentration, during the low flow pulsation phases as well as delayed contrast agent dynamics in the aneurysm (Fig 3). As opposed to the simultaneous 4D pc-fq velocity change, the dynamic signal of the outlet is slightly delayed with respect to the inlet of the model, since the contrast agent bolus needs time to travel through the model. The change of velocity, however, travels with the speed of sound through the medium. MPI and DSA even depicted the secondary low flow pulsation phase visible by the low broad local signal maxima between two consecutive high sharp contrast agent peaks. The dynamic MRI method with its 10 times lower temporal resolution depicted only the main high contrast agent peaks. However, MRI sufficiently detects the pump pulsation and the delayed contrast agent outflow from the aneurysm. Furthermore, the MRI signal of the aneurysm is higher as for the inlet or outlet, since the slower flow inside the aneurysm minimizes flow related MRI signal loss. Quantitative analysis of the dynamic imaging methods of Fig 3 are summarized in Table 2.

**Table 2:** Summary of dynamic measurement analysis (mean ± standard deviation).

	MRI	DSA	MPI
Bolus length:	2.96 s ± 0.08 s	2.9 s ± 0.03 s	3.05 ± 0.06 s
Pulsation rate:	80.3/s ± 6.7/s	76.2/s ± 3.6/s	74.0/s ± 2.7/s
Aneurysm contrast agent delay:	0.08 s ± 0.07 s	0.15 s ± 0.05 s	0.13 s ± 0.05 s

**Fig 4:**
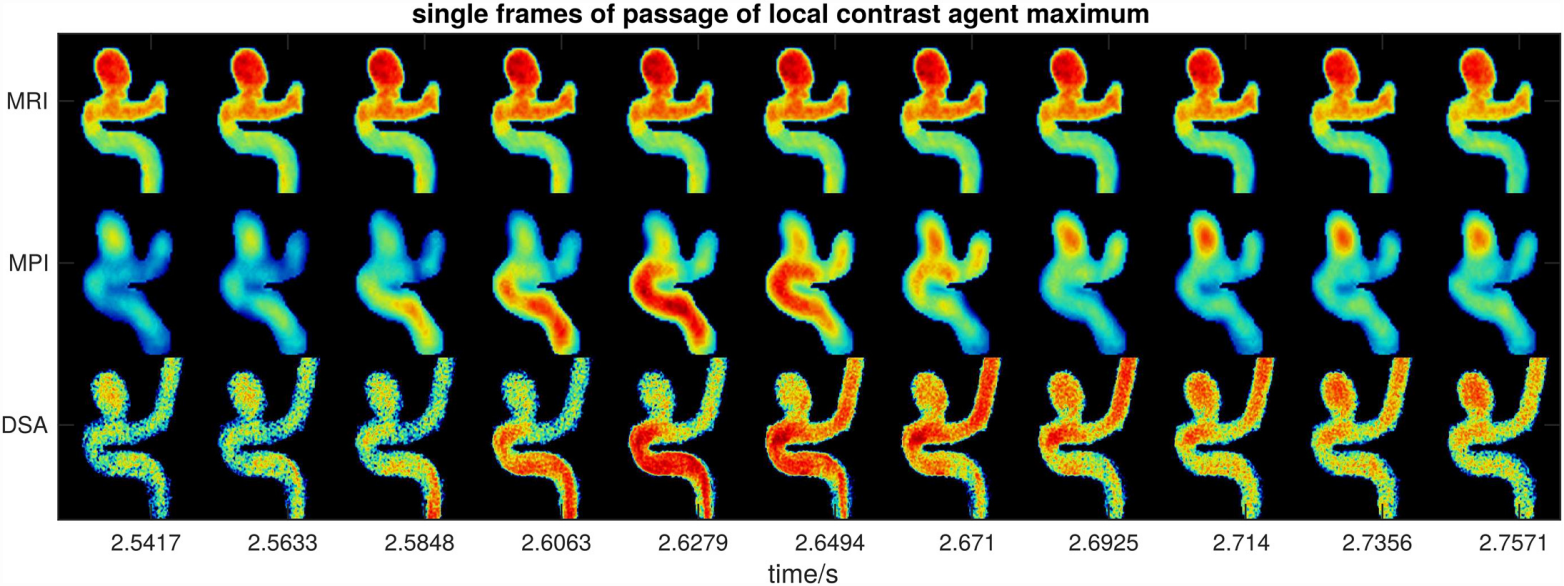
Multiple frames of dynamic MRI, MPI, and DSA measurements during the passage of a local maximum of contrast agent concentration (see time points 2.5 s-2.8 s in Fig 2). The passage of the contrast agent bolus through the main vessel of the model can be estimated in MPI and DSA, but not in MRI. A movie showing all frames of the dynamic scans is available as S3 Video.

Single frames around a local maximum contrast agent concentration allow depicting the contrast agent passage through the model for MPI and DSA but not for MRI (Fig 4). The front of the local maximum of the contrast agent concentration appears at time point 2.58 s and seems to have passed the model at time point 2.65 s. The distance through the model is approximately 4 cm resulting in an estimated flow velocity of 62 cm/s, which is in agreement with the 4D pc-fq measurements.

## Discussion

MPI is feasible to assess the hemodynamics of a realistic brain aneurysm model with high temporal detail. High frame rate DSA was also able to assess the contrast agent passage with high temporal detail, but only as a 2D projection and with high dose of ionizing radiation. Dynamic MRI, on the other hand, offers sufficient spatial resolution, but lacks proper temporal resolution to display the secondary flow pulsation. It was not possible to estimate the flow velocities based on the passage of the contrast agent through the model with the dynamic MRI method. However, the 4h long 4D phase contrast flow quantification MRI method allows for a nearly perfect depiction and characterization of the flow patterns of the aneurysm model, which is very helpful to better understand the observed contrast agent dynamics. Thus, the delayed contrast agent dynamics in the aneurysm are most likely caused by the vortex and lower flow velocities inside the aneurysm.

The periodic pulsation patterns of the dynamic imaging methods are caused by the modulation of the contrast agent concentration, due to the relatively slow injection of the contrast agent bolus into the pulsating carrier flow of the pump. During the short phase of low flow of the pump, the slowly but constantly injected contrast agent bolus is less diluted. On the other hand, during the phases of high flow of the pump pulsation there is increased dilution of the constantly injected contrast agent bolus. Such a modulation is strongly unwanted in common DSA diagnostics, where much higher injection rates are used to sufficiently and evenly fill the vascular system under examination. The commonly fast contrast bolus injections in DSA displace the pulsating carrier flow and sufficiently eliminate the unwanted modulation of the contrast agent concentration with the carrier flow pulsation.

The flow velocity estimation by visual tracking the front of the local contrast peak through the model is in agreement with an automated iterative optical flow based approach analyzing the blood flow along the centerline of the vessel of interest using dynamic DSA data [20]. Furthermore, a 2D optical flow based approach has also been suggested to assess intra-aneurysmal flow changes induced by flow-diverter stents [23]. However, the development and application of such methods to the MPI data has to be referred to future works. We carefully chose the flow and pulsation rate to match physiological conditions as they would be expected in a “healthy” patient for elective aneurysm treatment. Testing different flow and pulsation rates may be of interest for other clinical applications, like vascular occlusion or stenosis, but this is not the scope of our study regarding cerebral aneurysms. These tests will definitely be of interest for future studies with the scope of such applications.

The imaging parameters were optimized for fastest possible frame rate and subsequently available maximal spatial resolution of each imaging modality. Measurements with lower temporal resolution and subsequently higher spatial resolution or higher signal are possible, but will miss important temporal information for sufficient dynamic bolus imaging and tracking. Furthermore, the contrast agent concentrations for bolus injections for the different imaging modalities, were chosen after commercial availability (iodine and iron oxide based) and optimal signal delivery (Gadolinium based).

There are several potential clinical implications of our findings. First, once a human MPI scanner is available it will be an instant and powerful clinical tool for the in vivo assessment of the hemodynamics of intra cranial aneurysms with 4D high resolution without ionizing radiation as required by DSA. Second, additively manufactured real-sized and patient-specific aneurysm models are the key technology to translate powerful but yet clinically infeasible methods, like a 4 hours lasting scan of flow quantification MRI, into the clinic to the direct benefit of the patient, e.g., by investigating the flow patterns of the aneurysm with great detail and using this information for treatment planning. We also showed, that the already clinically available dynamic MRI and DSA methods were able to detect the delayed contrast agent dynamics of the aneurysm (Fig 3) and are, therefore, valid tools to assess and characterize the hemodynamics of aneurysms. However, the dynamic MRI method in human may have marked lower spatial and temporal resolution as demonstrated in the presented work and the dynamic DSA method may expose the patient to a considerably higher radiation dose. Cerebral aneurysms were chosen for our study, since a realistic cerebral aneurysm model from a previous study was already available [17]. We acknowledge that non-cerebral aneurysms would also be an equivalent testbed. MPI will add great value to the clinic in the context of cerebral aneurysms and other vascular pathologies. We expect, once MPI is available and greatly integrated into the clinical decision making process, it will be the method of choice before X-ray angiography.

A realistic aneurysm model was chosen, since we wanted to demonstrate and compare the dynamic imaging methods for a model geometry as it could appear in a patient. Using simple geometries for the comparisons may be of technical interest, but has an even lower clinical impact than using realistic geometries. We do also not expect any effect on the comparison of the different dynamic imaging modalities, since the exact flow field is known due to the 4D phase contrast flow quantitative MRI measurement. The 4D pc-fq was very helpful in interpreting the delayed contrast agent dynamics of the aneurysm due to the observed vortex inside of the aneurysm.

The main limitation of our study is that we so far investigated only one particular aneurysm with a very characteristic location and morphology, which may limit the generality of our findings. However, since the purpose of this work was to proof the concept of assessing the hemodynamics of a realistic aneurysm model with MPI and to compare the results with MRI and DSA, we expect that this will be also true for other aneurysm models with different morphologies. Concerning the rigid material and layered structure of the model, we do not expect that any residual surface structure will affect the comparison of the different imaging techniques, since the same model was used for all measurements and due to the much smaller size of these surface structures compared to the lumen of the model. Flexible materials, currently investigated for feasibility at our clinic, are of interest for simulating potential complications, like aneurysm ruptures during intervention, but were not readily available for our study. However, the choice of the rigid model does not impact the suitability of the testbed or clinical relevance of our study. A further limitation concerns the non-physiologic pulsation curve of the used peristaltic pump. The pump pulsation profile is characterized by a short low flow phase and a long period of high flow velocities as opposed to physiological pulsation with a single short peak of high flow and a longer period of lower flow velocities. Thus, adjusting the pump to a physiologically reasonable flow rate may cause a lower maximum flow velocity as for a physiological pulsation profile. However, higher maximum flow velocities may easily be obtained by increasing the flow rate of the pump above physiological flow rates.

## Conclusions

MPI is a promising dynamic imaging method for the 4D high resolution assessment of the hemodynamics of a realistic intracranial aneurysm model. Thus, human MPI scanners, once available, could become a powerful clinical tool for the in vivo assessment of aneurysm hemodynamics. On the other hand, real-sized patient specific additively manufactured aneurysm models are a potent translational tool to obtain important information, e.g. perfect depiction of aneurysm hemodynamics assessed with very long 4D pc-fq MRI, which is not possible to obtain in vivo. Already clinically available dynamic MRI and DSA are also proper but partly limited tools to assess and characterize the hemodynamics of aneurysms.

## Supporting Information

S1 Fig Peristaltic pump. The rotation of the pump (35 U/min) is set to half of the desired pulsation rate (70/min), since the two rollers (yellow arrow) of the rotor transmit a pulsation rate which is twice the rotation rate. The flow rate is adjusted empirically by setting the gap between the rollers and the inner wall of the pump (thumb wheel, blue arrow). A pneumatic sensor was placed underneath the tube (green arrow) to trigger and synchronize the 4D phase contrast flow quantification (4D pc-fq) MRI scan with pump pulsation.
(TIF)

S1 Video False color visualization of the phases of the 4D pc-fq MRI during one pulsation cycle. (AVI)

S2 Video Vector field visualization of the phases of the 4D pc-fq MRI during one pulsation cycle. (AVI)

S3 Video False color visualization of the dynamic imaging methods MRI during bolus passage. (AVI)
