# Supplementary figures and images for: Magnetic Particle Imaging for High Temporal Resolution Assessment of Aneurysm Hemodynamics
Source: PLoS One. 2016 Aug 5;11(8):e0160097. doi: 10.1371/journal.pone.0160097 (PMC4975468; doi:10.1371/journal.pone.0160097)

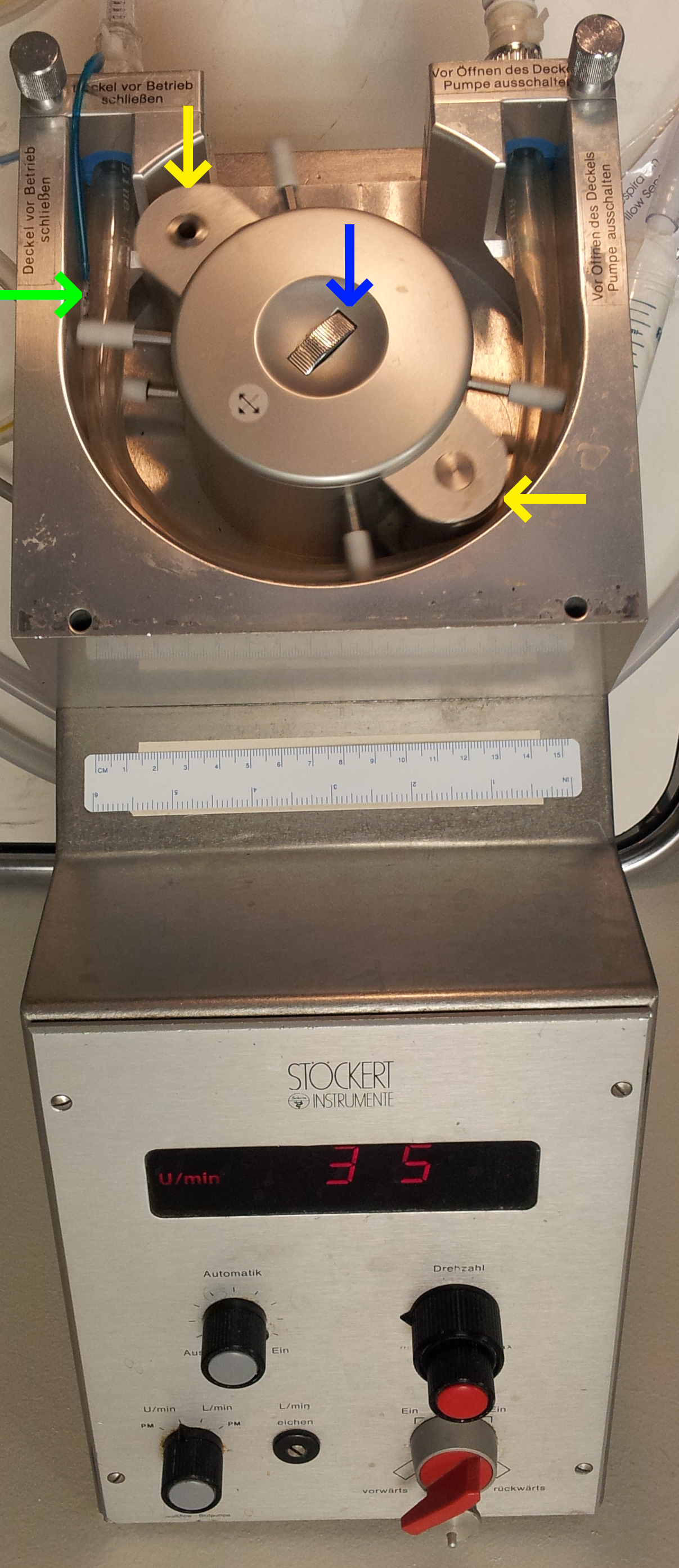

Supplement: S1 Fig — The rotation of the pump (35 U/min) is set to half of the desired pulsation rate (70/min), since the two rollers (yellow arrow) of the rotor transmit a pulsation rate which is twice the rotation rate. The flow rate is adjusted empirically by setting the gap between the rollers and the inner wall of the pump (thumb wheel, blue arrow). A pneumatic sensor was placed underneath the tube (green arrow) to trigger and synchronize the 4D phase contrast flow quantification (4D pc-fq) MRI scan with pump pulsation. (TIF) [file pone.0160097.s001.tif]
